# Supplementary material for: Anticoagulation Control and Major Adverse Clinical Events in Patients with Operated Valvular Heart Disease with and without Atrial Fibrillation Receiving Vitamin K Antagonists
Source: J Clin Med. 2023 Feb 1;12(3):1141. doi: 10.3390/jcm12031141 (PMC9917463; doi:10.3390/jcm12031141)
Supplement: Supplementary file 1 [file jcm-12-01141-s001.zip › jcm-2148589-supplementary.pdf]

SUPPELEMENT

Table S1: Adverse clinical outcome vs. TTR among patients with operated valvular heart disease, with and without AF

N (%)	TTR<70% N=358	TTR≥70% N=98	p-value	TTR<65% N=310	TTR≥65% N=146	p-value
**Stroke/TIA/SE**	23 (6.4)	2 (2.0)	0.13	20 (6.5)	5 (3.4)	0.19
**Bleeding***	72 (20.1)	13 (13.3)	0.12	65 (21.0)	20 (13.7)	0.06
**CV hospitalisation**	65 (18.2)	13 (13.3)	0.26	58 (18.7)	20 (13.7)	0.19
**All-cause death**	47 (13.1)	4 (4.1)	**0.011**	44 (14.2)	7 (4.8)	**0.003**
**≥1 MACE†**	153 (42.7)	27 (27.6)	**0.006**	138 (44.5)	42 (28.8)	**0.001**

Cardiovascular hospitalisation: a hospitalisation with a cardiovascular cause: i) heart failure, MI, new angina, non-fatal cardiac arrest, ventricular arrhythmia, uncontrolled AF/atrial flutter, supraventricular arrhythmia, ii) valve surgery, CABG surgery, PTCA surgery, pacemaker/ICD insertion, carotid endarterectomy, peripheral angioplasty/surgery, limb amputation AND as recorded in patient's medical documents; DVT – Deep Vein Thrombosis; Major Bleeding – ISTH Major Bleeding: fatal bleeding and/or symptomatic bleeding in a critical area or organ, such as intracranial, intraspinal, intraocular, retroperitoneal, intraarticular or pericardial, or intramuscular with compartment syndrome and/or bleeding causing a fall in haemoglobin level of 2 g/dL (1.24 mmol/L) or more, or leading to transfusion of two or more units of whole blood or red cells; Clinically relevant non-major bleeding (CRNMB): clinically overt bleeding not satisfying the criteria for major bleeding but meet at least one of the 3 criteria: i) leading to hospitalisation or increased level of care, ii) requiring medical intervention by healthcare professional and iii) prompting face to face evaluation; SE: systemic embolism; TIA: transient ischemic attack; VTE: venous thromboembolism.

Definitions of outcomes

Major adverse clinical events (MACE)

Major adverse clinical events (MACE) of interest were stroke/transient ischemic attack (TIA)/systemic embolism, bleeding (combination of major bleed and clinically relevant non major bleed), cardiovascular (CV) hospitalisation, death and a composite (≥ 1) of any MACE.

Stroke was defined as any focal neurologic deficit, from a non-traumatic cause, lasting at least 24 hours and further categorized as ischaemic (with or without haemorrhagic transformation), haemorrhagic, or of uncertain type (where brain imaging or autopsy was not performed). A thromboembolic event outside the brain, retina, heart or lungs was classified as systemic embolism. Stroke and systemic embolism were combined as thromboembolic events (TE).

Major bleeding was classified according to the ISTH criteria [16] as: fatal bleeding, and/or symptomatic bleeding in a critical area or organ, such as intracranial, intraspinal, intraocular, retroperitoneal, intra-articular or pericardial, or intramuscular with compartment syndrome, and/or bleeding causing a fall in haemoglobin level of 20 g/L or more, or leading to transfusion of two or more units of whole blood or red cells) [16]. Clinically relevant non-major bleeding (CRNMB) was defined as clinically overt bleeding that did not satisfy the criteria for major bleeding and that led to hospital admission, physician-guided medical or surgical treatment, or a change in antithrombotic therapy [16]. Major bleeding and CRNMB were combined as bleeding events.

Cardiovascular hospitalisation was defined as a hospitalisation with a cardiovascular cause: i) heart failure, myocardial infarction (MI), new angina, non-fatal cardiac arrest, ventricular arrhythmia, uncontrolled AF/atrial flutter, supraventricular arrhythmia, ii) valve surgery, coronary artery bypass graft (CABG) surgery, percutaneous transluminal coronary angioplasty (PTCA) surgery, pacemaker/ implantable cardioverter-defibrillator (ICD) insertion, carotid endarterectomy, peripheral angioplasty/surgery, limb amputation [17], and recorded in patient's EHRs.

In this study, the cause of death was specified as CV death when specific information was available. Where cause of death was unavailable, death was classified as all-cause death.
